# Supplementary material for: HIVprotI: an integrated web based platform for prediction and design of HIV proteins inhibitors
Source: J Cheminform. 2018 Mar 9;10:12. doi: 10.1186/s13321-018-0266-y (PMC5845081; doi:10.1186/s13321-018-0266-y)
Supplement: Supplementary file 2 — Additional file 2. Source code of HIVProtI web server. [file 13321_2018_266_MOESM2_ESM.zip › HIVprotI_Source-code/hivprotI/weka-3-6-6/doc/weka/gui/sql/event/package-summary.html]

weka.gui.sql.event


---


|  |  |  |
| --- | --- | --- |
| **Overview** \| **Package** \| Class \| **Tree** \| **Deprecated** \| **Index** \| **Help** \| **Weka's home** | |  |
| **PREV PACKAGE**   **NEXT PACKAGE** | **FRAMES**    **NO FRAMES**     **All Classes** |  |


---

## Package weka.gui.sql.event

| **Interface Summary** | |
| --- | --- |
| **ConnectionListener** | A listener for connect/disconnect events. |
| **HistoryChangedListener** | A listener for changes in a history. |
| **QueryExecuteListener** | A listener for executing queries. |
| **ResultChangedListener** | A listener that is notified if another Result is activated in the ResultPanel. |

| **Class Summary** | |
| --- | --- |
| **ConnectionEvent** | An event that is generated when a connection is established or dropped. |
| **HistoryChangedEvent** | An event that is generated when a history is modified. |
| **Messages** | Messages. |
| **QueryExecuteEvent** | An event that is generated when a query is executed. |
| **ResultChangedEvent** | An event that is generated when a different Result is activated in the ResultPanel. |

---


|  |  |  |
| --- | --- | --- |
| **Overview** \| **Package** \| Class \| **Tree** \| **Deprecated** \| **Index** \| **Help** \| **Weka's home** | |  |
| **PREV PACKAGE**   **NEXT PACKAGE** | **FRAMES**    **NO FRAMES**     **All Classes** |  |


---
